# Glymphatic System and Subsidiary Pathways Drive Nanoparticles Away from the Brain

**DOI:** 10.34133/2022/9847612

**Published:** 2022-03-15

**Authors:** Rui Liu, Wenfeng Jia, Yushan Wang, Chuan Hu, Wenqi Yu, Yuan Huang, Ling Wang, Huile Gao

**Affiliations:** Key Laboratory of Drug-Targeting and Drug Delivery System of the Education Ministry and Sichuan Province, Sichuan Engineering Laboratory for Plant-Sourced Drug and Sichuan Research Center for Drug Precision Industrial Technology, West China School of Pharmacy, Sichuan University, Chengdu 610041, China

## Abstract

Although drug delivery systems (DDS) are efficient in brain delivery, they face failure in clinical settings due to their potential toxicity to the central nervous system. Little is known about where the DDS will go after brain delivery, and no specific elimination route that shares a passage with DDS has been verified. Hence, identifying harmless DDS for brain delivery and determining their fate there would strongly contribute to their clinical translation. In this study, we investigated nonreactive gold nanoclusters, which can deliver into the brain, to determine the elimination route of DDS. Subsequently, nanoclusters in the brain were systemically tracked and were found to be critically drained by the glymphatic system from the blood vessel basement membrane to periphery circulations (77.8 ± 23.2% and 43.7 ± 23.4% contribution). Furthermore, the nanoclusters could be actively transported across the blood-brain barrier (BBB) by exosomes (30.5 ± 27.3% and 29.2 ± 7.1% contribution). In addition, microglia promoted glymphatic drainage and passage across the BBB. The simultaneous work of the glymphatic system, BBB, and microglia revealed the fate of gold nanoclusters for brain delivery and provided a basis for further brain-delivery DDS.

## 1. Introduction

In order to ameliorate poor nidus drug delivery in central nervous system (CNS) diseases [1], drug delivery systems (DDS) have been extensively established to conquer the blood-brain barrier (BBB) [2–14]. The BBB, which is made up of endothelial cells with tight junctions, basement membrane, and astrocytic end-feet [15, 16], is a major obstacle for therapeutic agents [17]. These DDS have similar nanometer sizes and contain nanomaterials to deliver therapeutic agents and motifs to cross the BBB. In addition, the motifs can further introduce the DDS to the nidus [5, 6, 18–20]. While many targeting strategies, including DDS for CNS diseases, have been proven to be effective, brain delivery DDS has a failure rate of 100% in clinical translation [21]. Taking this into account, DDS for CNS delivery are generally more restricted in certain aspects, such as biocompatibility, compared to periphery applications [22], as a consequence of rash disruption to homeostatic CNS.

Despite the importance of biocompatibility and safety for CNS-targeting DDS, almost all studies focused on how to deliver the DDS into the CNS while ignoring the fate of DDS after delivery. Although certain DDS are endogenous, there remain unknown issues that can arise from longtime accumulation if they are not excreted, with amyloid-*β* (A*β*) a good example of this theory [23, 24]. Exogenous DDS, which are unlikely to be degraded by CNS, have even worse complications. Therefore, it is essential to identify the brain elimination routes of DDS in order to benefit clinical translation. At present, the elimination of DDS from the brain via a specific route has yet to be proven. There is no verified specific elimination route that involves nano-sized DDS, which complicates the current clarification and feedback for future investigations on brain elimination. This existing predicament will not be resolved until evidence of brain elimination of DDS is validated, at which point new, consistent criteria for judging and selecting brain delivery DDS with rational elimination will be established.

Since the cerebrospinal fluid (CSF) and lymphatics in the nasal mucosa and dura mater are connected [25], the major issue for brain elimination is transportation from the brain parenchyma to the CSF [26]. Although the solutes in interstitial fluid (ISF) can mix with the CSF [26] in other ways, the most probable route that DDS may take is the glymphatic system, which is an intracerebral lymphatics-like system that drains the solutes in the deep brain along the capillary and arterial basement membrane for intramural peri-arterial drainage (IPAD) [25, 27–29]. Tracers, such as dyes and A*β*, are reported to clear mainly through the glymphatic system [25, 30]. However, 15 nm gold nanoparticles were unable to enter the glymphatic system and were located beside the blood vessels instead of inside the basement membrane [25]. Although glymphatic drainage of solid lipid nanoparticles (SLN) around 100 nm has been reported [31], we believe that proving glymphatic drainage with only vein-SLN colocation, which was also observed with 15 nm gold nanoparticles, is insufficient. Furthermore, the basement membrane associated with the glymphatic system appears to be too narrow for 100 nm nanoparticles to pass through [25]. Therefore, more information on both the glymphatic system and its suitable DDS properties is required. Moreover, DDS can cross the BBB for elimination, similar to A*β* [32], which may involve a similar mechanism of active transport or other unknown ones, but more evidence is required to support this hypothesis.

Gold nanoclusters (AuNCs) are ultrasmall gold nanoparticles with a nonreactive Au core and an endogenous shell [33, 34]. Importantly, AuNCs have high potential for brain delivery due to their superior properties, such as rapid renal clearance, efficient drug delivery, and convenient marker detection [35–40]. Nonetheless, given the current state of general DDS, the unknown elimination route remains a huge challenge to their application in CNS, strongly limiting their clinical translation.

In this study, we investigated the elimination routes used by nano-sized DDS to leave the CNS using selected bovine serum albumin (BSA) and glutathione (GSH) coated AuNCs (AuNC@BSA, AuNC@GSH). As the representations of general DDS with excellent CNS delivery, the behavior of AuNCs in the brain would reveal the fate of DDS post brain delivery. After the elimination routes have been established, three major concerns should be addressed: 1) could the nano-sized DDS for brain delivery leave the CNS to reduce undesired effect; 2) was there a well-proven intracerebral pathway where the DDS could sufficiently enter the periphery; 3) which specific route and explicit mechanism did the AuNCs use to leave the brain. The new insights would undoubtedly be beneficial for brain delivery DDS, allowing their applications to be scaled to new heights.

## 2. Results

### 2.1. Particle Characterization

**Figure 1:**
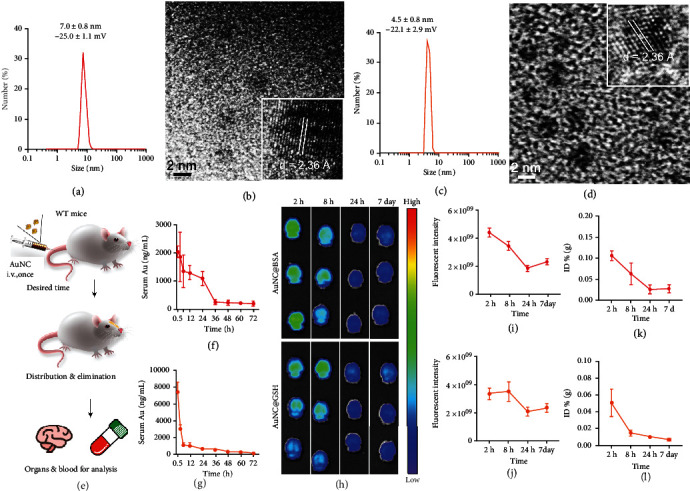
DLS characterization of AuNC@BSA (a) and AuNC@GSH (c), and their HRTEM images (b, d), respectively. (e) The illustration of experiment procedure of systemic distribution and elimination, the two AuNCs were i.v. injected into wild type (WT) mice and obtained the samples at different time points for measurements. (f, g) The AuNCs in circulation were evaluated by ICP-MS. (h) The FLI of brains at multiple time points. The color scale ranges from 1 × 10^8^ to 6 × 10^8^, and unit is (p/sec/cm^2^/sr)/(*μ*W/cm^2^). The AuNCs in brains were semi-quantified by FLI (i, j) and quantified by ICP-MS (k, l). In the whole picture, the AuNC@BSA-associated statistic data showed in red curves, while the orange ones for AuNC@GSH, the same below.

Both AuNCs were obtained using the one-step approaches that were previously described [33, 34]. The dynamic light scattering (DLS) results showed that the hydrodynamic diameter of AuNC@BSA was 7.0 ± 0.8 nm (Figure 1(a)), which was slightly higher than that of AuNC@GSH (4.5 ± 0.8 nm, Figure 1(c)). In addition, both were negatively charged, with zeta potentials greater than -20 mV. Based on the captures of the high-resolution transmission electron microscope (HRTEM), both AuNCs contained 2 nm Au cores (Figure 1(b), 1(d)), which was consistent with previous reports [41–45]. Their interplanar spacing of the crystal lattices was 2.36 Å, which was consistent with the data of Au (111) in the face-centered cubic crystal.

### 2.2. Systemic Distribution and Elimination after Intravenous Administration

BALB/c mice were used to evaluate the systemic distribution of intravenous (i.v.) AuNCs (Figure 1(e)). Fluorescence imaging (FLI, Figure S1) and the associated fluorescence intensity (Figure S2) validated that both AuNCs were critically eliminated from the kidney and liver. Quantification of Au content (Figure S3) by inductively coupled plasma mass spectrometry (ICP-MS) revealed similar results to the FLI. The Au content in peripheral circulation also reduced rapidly (Figure 1(f), 1(g)), synergistically indicating that AuNCs were efficiently eliminated in the periphery elimination [35–37]. Following that, FLI verified that there was fluorescence in the brain at 2 h (Figure 1(h)), which started to vanish thereafter. The fluorescence intensity (Figure 1(i), 1(j)) and Au contents (Figure 1(k), 1(l)) confirmed that AuNCs entered the brain and were almost completely cleared from the central nervous system (CNS) within 24 h.

**Figure 2:**
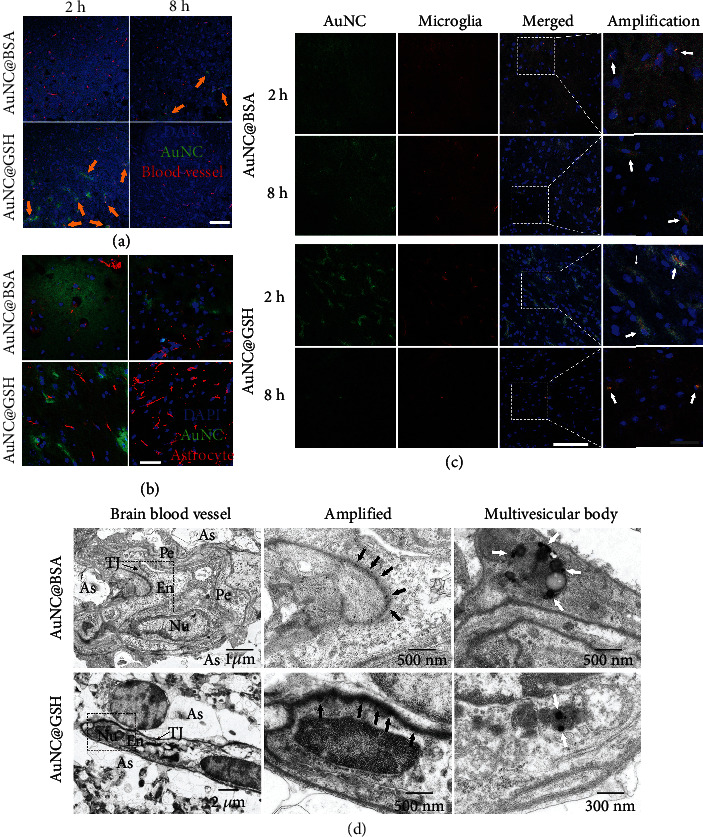
(a) Confocal imaging of AuNCs (green), nuclei (blue), and anti-CD31 antibody-stained microglia (red), white bar represents 50 *μ*m. The orange arrows indicate the co-localization of the endothelia cells and AuNCs. (b) Confocal imaging of AuNCs (green), nuclei (blue), and anti-GFAP antibody-stained astrocytes (red), white bar represents 50 *μ*m. (c) Confocal imaging of AuNCs (green), nuclei (blue), and anti-TMEM-119 antibody-stained endothelia cells (red), white bar represent 100 *μ*m, grey bar represents 30 *μ*m. The white arrows indicate the co-localization of the microglia and AuNCs. (d) The TEM images of AuNCs in brains, the black arrows indicate where the AuNCs lie, while the white arrows show the former exosomes containing AuNCs in multivesicular bodies. En = endothelia cells, As = astrocytes end-feet, Nu = the nuclei of endothelia cells, TJ = tight junction of endothelia cells, Pe = pericytes.

Similar to the bio-distribution results, AuNC@BSA (Figure S4) and AuNC@GSH (Figure S5) rapidly accumulated and gradually decreased in tissues. Furthermore, both AuNCs displayed polarized sub-organ distribution, especially in the brain. Although AuNCs in the peripheral organs were mainly found with epithelial cells, those in the brain tended to form aggregates (seen in AuNC@BSA at 8 h and AuNC@GSH at 2 h), which were cleared as time passed. The AuNC aggregates were found to be surrounding the endothelium of brain vessels by immunofluorescence tracking, (Figure 2(a)), but were not well collocated. The periphery AuNCs did not associate with blood vessels (Figure S6), indicating that the aggregates were brain-specific, and possibly a result of brain elimination. Since astrocytic end-feet are a part of the BBB and surround endothelium, their collocation with AuNCs was evaluated. However, poor colocation was discovered (Figure 2(b)), indicating that they probably had limited contribution. Microglia, which are the equivalent macrophages in CNS, showed strong collocation with the AuNC aggregates (Figure 2(c)), suggesting that microglia may potentially work with AuNCs in the CNS.

Based on TEM imaging, there were abundant AuNCs in the basement membrane between the endothelium and astrocytic end-feet (Figure 2(d)). This finding was consistent with recent reports on glymphatic drainage [25], similar to peripheral lymphatic drainage, and allowing ISF to drain along the basement membrane to leave the CNS. Moreover, AuNCs were trapped in multivesicular bodies, which were producing exosomes around 100 nm, in the cytoplasm of the endothelium [46, 47]. This phenomenon suggested that exosomes could be the carriers of AuNCs across BBB.

### 2.3. Glymphatic Drainage Is Responsible for the Elimination of Bulk AuNCs

**Figure 3:**
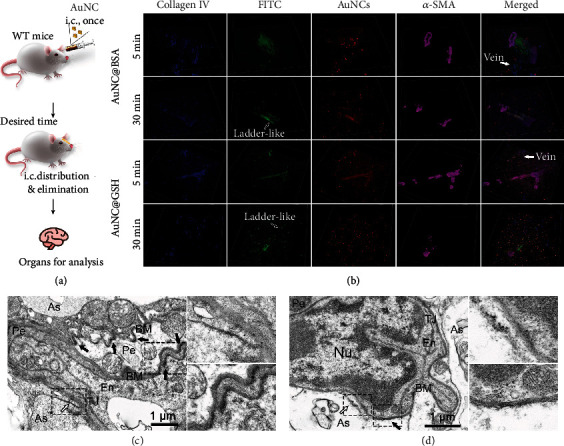
(a) Illustration of i.c. injection of bulk AuNCs for separate investigation on the elimination. The brains were obtained at 5 or 30 min post injection to capture the dominant excreting phase. (b) Three dimensions (3D) reconstructions of confocal images to show the colocalization of AuNCs (green and red), collagen IV (blue) and *α*-SMA (pink). The images were captured every 2.5 *μ*m for about 50 *μ*m before 3D reconstruction. The hollow arrows indicate the ladder-like structure formed by AuNCs, while the solid arrows indicate the capillary veins without smooth muscle. (c-d) The TEM images of the WT mice brain received i.c. administration of AuNC@BSA and AuNC@GSH (30 min), respectively. The black arrows indicate the AuNCs in the basement membrane for glymphatic drainage, while the white arrows indicate the astrocytes end-feet, where the AuNCs entered the glymphatic system through high-expressed AQP4 protein. En = endothelia cells, As = astrocytes end-feet, Nu = the nuclei of endothelia cells, TJ = tight junction of endothelia cells, Pe = pericytes, BM = basement membrane.

The BALB/c mice were used to monitor the elimination of intracerebral (i.c.) AuNCs (Figure 3(a)). According to the labeling of collagen IV in the basement membrane and smooth muscle actin *α* (*α*-SMA) in arterial smooth muscles, the AuNCs were visible traveling along the basement membrane that was located between the smooth muscles in the tunica media of arteries (Figure 3(b)), which was the pathway of the glymphatic system, followed by proceeding to the intramural peri-arterial drainage (IPAD) [25, 27, 28]. At 30 min post-injection, the AuNCs signal displayed a ladder-like structure, which has been reported to be a characteristic phenomenon of the glymphatic system and formed by the artery pulse [28, 30]. Furthermore, in the images 5 min post-injection (clearer in the videos), there were AuNCs in the basement membrane without *α*-SMA coating, which belonged to the capillaries. In the TEM images, there was an abundance of i.c. AuNCs in the basement membrane (Figure 3(c), 3(d)). Furthermore, there were AuNCs located at the astrocytic end-feet, which had an abundance of aquaporin 4 protein (AQP4) to ensure efficient water exchange in the CNS [48] and served as the glymphatic drainage gateway [31, 49]. Despite our best efforts, we were unable to capture the brain artery using TEM due to the limited area of the ultrathin slice and the rare distribution of the artery.

### 2.4. Cerebrospinal Fluid Propelled the Glymphatic Drainage

**Figure 4:**
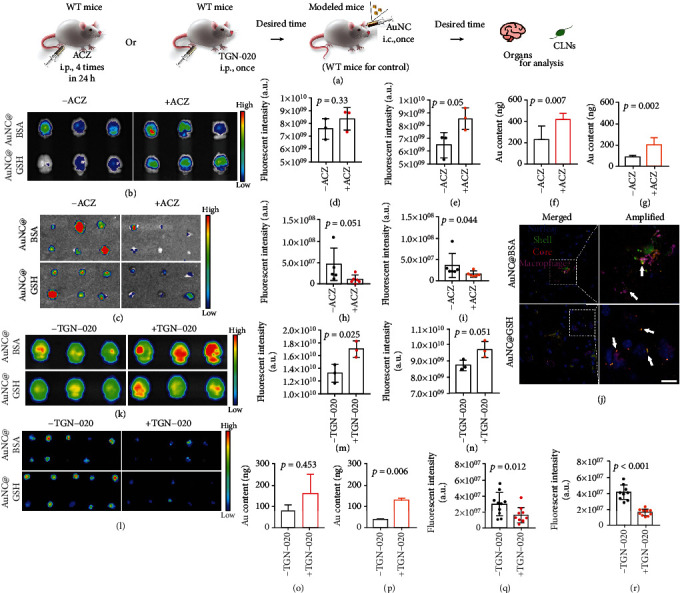
(a) Illustration of the experiment procedure for the pathways investigations associated with glymphatic drainage. The WT mice were pretreated with ACZ or TGN-020 for a certain period to generate modeled mice, then received i.c. injection of AuNCs and their brains with CLNs were collected for analysis. (b) The FLI of brains with AuNCs distribution after ACZ pretreatment, the color scale ranges from 5.0 × 10^8^ to 1.0 × 10^9^. (c) The FLI of the CLNs, the color scale ranges from 2.0 × 10^7^ to 5.0 × 10^7^. The semi-quantification of the fluorescent intensity in brains for AuNC@BSA (d) and AuNC@GSH (e), and their Au contents in brain quantified by the ICP-MS (f, g, respectively). The fluorescent intensity in CLNs were also semi-quantified (h, i). (j) The confocal imaging of AuNCs (green and red) internalized by peri-artery macrophages (pink), with blue indicates the nuclei. The white bar represents 5 *μ*m. The white arrows indicate the post-internalization AuNCs in the macrophages. (k) The FLI of brains with AuNCs distribution after TGN-020 pretreatment, the color scale ranges from 1.0 × 10^8^ to 1.6 × 10^9^ for AuNC@BSA, and 1.0 × 10^8^ to 1.0 × 10^9^ for AuNC@GSH. (l) The FLI of the CLNs, the color scale ranges from 3 × 10^7^ to 7 × 10^7^. The semi-quantification of fluorescent intensity (m, n) and ICP-MS quantification (o, p) of AuNCs in brains, and the fluorescent intensity in CLNs containing AuNCs (q, r). The unit for fluorescent imaging is (p/sec/cm^2^/sr)/(*μ*W/cm^2^).

The factors that affected the glymphatic drainage of AuNCs were detected in modeled mice that had been pretreated with acetazolamide (ACZ, a CSF production inhibitor [50]) (Figure 4(a)). The inhibited glymphatic drainage in ACZ-treated mice was confirmed by FITC-inulin (fluorescein isothiocyanate inulin), which could not be intracellularly transported and participate in biochemical processes in the animal body. Based on fluorescence imaging and quantification, ACZ attenuated inulin that was eliminated from mouse brains and passed through cervical lymphatic nodes (CLNs), with an inhibition rate of 25.4 ± 0.8% (Figure S7). Subsequently, i.c. AuNCs were significantly retained more when compared to WT mice (Figure 4(b)). Correspondingly, the AuNCs signals in CLNs were significantly reduced due to pretreatment with ACZ (Figure 4(c)). The fluorescence intensity (Figure 4(d), 4(e)) and Au contents (Figure 4(f), 4(g)) in the brain confirmed the significant differences between the WT and ACZ-treated mice, as well as the fluorescence intensity in CLNs (Figure 4(h), 4(i)). The inhibition rate of AuNC@BSA and AuNC@GSH eliminated from the brain was determined to be 19.7 ± 5.9% and 11.1 ± 5.9%, respectively. Based on the route inhibition rate (25.4 ± 0.8%), brain elimination of AuNC@BSA and AuNC@GSH due to glymphatic drainage was 77.8 ± 23.2% and 43.7 ± 23.4%, respectively (Table S1). According to the labeling of peri-artery macrophages in the artery wall [51], we found that macrophages exhibited significant phagocytosis on AuNCs (Figure 4(j)), which was consistent with previous findings on glymphatic drainage [30]. After ACZ treatment, the uptake of macrophages was significantly weakened (Figure S8). These findings provided strong evidence that the CSF significantly promoted glymphatic drainage of AuNCs, allowing them to efficiently return to peripheral circulation via CLNs.

### 2.5. Aquaporin 4 Protein Represents a Gateway of the Glymphatic Drainage

Due to the consistency of our TEM results and previous findings [31, 48], the focus was shifted to the AQP4 water channel. When mice were pretreated with TGN-020 (Figure 4(a)), which is an AQP4 blocker [48, 52], there was a significant accumulation of AuNCs in their brains when compared to WT mice (Figure 4(k)). The corresponding signal in CLNs also showed a significant reduction (Figure 4(l)). After TGN-020 was inhibited, the fluorescence intensity (Figure 4(m), 4(n)) and Au contents (Figure 4(o), 4(p)) in the brain, as well as the fluorescence intensity in CLNs (Figure 4(q), 4(r)), displayed slower elimination of AuNCs. Immunofluorescence imaging further confirmed phagocytosis of AuNCs by peri-artery macrophages (Figure S9). Therefore, these findings demonstrated that the AQP4 water channel provided AuNCs with access to the glymphatic drainage, which was consistent with previous findings on AQP4 and the glymphatic system [48, 52].

### 2.6. Crossing the BBB Represents a Way for Clearing AuNCs from the Brain

**Figure 5:**
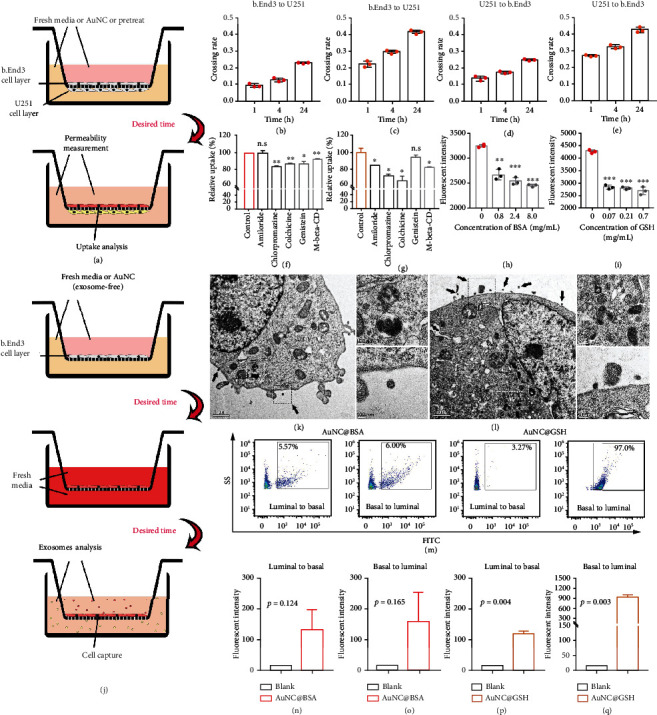
(a) Illustration of the BBB model construction in transwell chamber, the medium and cells were subjected to permeability or uptake analysis after incubation with the AuNCs. The permeability of AuNC@BSA (b) and AuNC@GSH (c) from bEnd.3 to U251 cells, and that of the two AuNCs from U251 to bEnd.3 cells (d, e, respectively). The results of uptake inhibition evaluated the uptake mechanisms of AuNC@BSA (f) and AuNC@GSH (g). According to literatures, amiloride inhibits the Na-K-ATPase to block macropinocytosis, chlorpromazine blocks clathrin-mediated endocytosis, genistein blocks caveolin-mediated endocytosis, and methyl-*β*-cyclodextrin (M-beta-CD) inhibits both lipid raft and caveolin-mediated endocytosis, colchicine works on macropinocytosis, clathrin-mediated endocytosis and others. The competitive inhibition by free BSA or GSH on the uptake of AuNC@BSA (h) and AuNC@GSH (i). (j) The illustration of approach that obtained exosomes and exosomes-secretion endothelia cells by using transwell chamber. (k-l) The TEM images of endothelia cells with secreted exosomes containing AuNC@BSA and AuNC@GSH, respectively. The black arrows indicate the exosomes. The images of post-phagocytosis multivesicular bodies and exosomes were amplified. (m) The signals of AuNCs in exosomes were analyzed by the nano-flow cytometry. The fluorescent intensity of FITC revealed the amounts of AuNCs. (n-q) The FITC fluorescent intensity of exosomes increased after the transport of the AuNCs.

Crossing the BBB was the only way for i.v. AuNCs to enter the brain, which may also contribute to the elimination of AuNCs. However, to our knowledge, only much smaller endogenous components, such as A*β*, can be eliminated via the BBB [32]. Therefore, it remained a challenge for AuNCs to be eliminated across the BBB. The intact static BBB model (bEnd.3/U251, Figure 5(a)) in transwell chambers revealed the permeability of both AuNCs in the direction of bEnd.3 to U251 cells (Figure 5(b), 5(c)), successfully simulating AuNCs entering the brain. Importantly, AuNCs also exhibited slightly stronger permeability in the direction of U251 to bEnd.3 cells (Figure 5(d), 5(e)), indicating that AuNCs were eliminated from the CNS. It was not possible for AuNCs to passively cross the tight junction between the endothelium of the BBB [15, 16]. Therefore, active transport should take charge of the AuNCs passage.

Chlorpromazine and genistein significantly reduced the uptake of AuNC@BSA, indicating that the caveolin-mediated and clathrin-mediated endocytosis were its major mechanisms, while amiloride-involved macropinocytosis had no contribution (Figure 5(f)). Similarly, the uptake mechanisms of AuNC@GSH were mainly clathrin-mediated endocytosis and macropinocytosis, instead of caveolin-mediated endocytosis (Figure 5(g)). In addition, lipid raft played a moderate role in the uptake of both AuNCs. The specific uptake of the AuNCs primarily relied on a receptor or transporter to trigger the above mechanisms. Subsequent competitive inhibition assays confirmed that the receptor or transporter of BSA or GSH, which were reported as secreted protein acidic and rich in cysteine (SPARC), glycoprotein 60 (gp60), and GSH transporter (GSHT) [53, 54], played key roles in the procedures (Figure 5(h), 5(i)).

Except for endocytosis, exocytosis on the other side of the endothelium remained important for intact BBB passage [20]. Although exocytosis of intracellular cargo relied on general vesicles derived from the membrane system [55, 56], there were dominant multivesicular bodies in TEM images of mouse brains receiving i.v. AuNCs, suggesting that exosomes, instead of general vesicles, may take charge of AuNCs exocytosis. The extracellular uniform sphere and membrane-coating property of exosomes would fuse with other cellular membranes to facilitate cargo transport [57]. Endothelial cells incubated with AuNCs in transwell chambers were subjected to TEM capture and exosome analysis (Figure 5(j)). The incubation triggered abundant secretory exosomes containing AuNCs in the dark signal, as seen in the TEM view (Figure 5(k), 5(l)). Furthermore, multivesicular bodies with AuNCs were also visible in the cytoplasm. The collected samples were verified as exosomes with apparent CD9 expression (Figure S10), with the practicability of exosomes analysis confirmed using the nano-flow cytometer (nano-FCM). Subsequently, by monitoring the unstained exosomes, distinguished groups showing stronger AuNCs signals were identified (Figure 5(m)). The exosomes secreted in both sides of endothelium showed comparable contents of AuNC@BSA (4.1 ± 2.7% and 3.7 ± 2.68%, respectively), while there was a big disparity in AuNC@GSH contents (97.7 ± 1.0% and 2.0 ± 1.8%). The average signal in the exosomes was strongly elevated with AuNCs treatment (Figure 5(n)-5(q)). Therefore, these observations and analyses validated that exosomes were the carriers of AuNCs leaving the endothelium.

**Figure 6:**
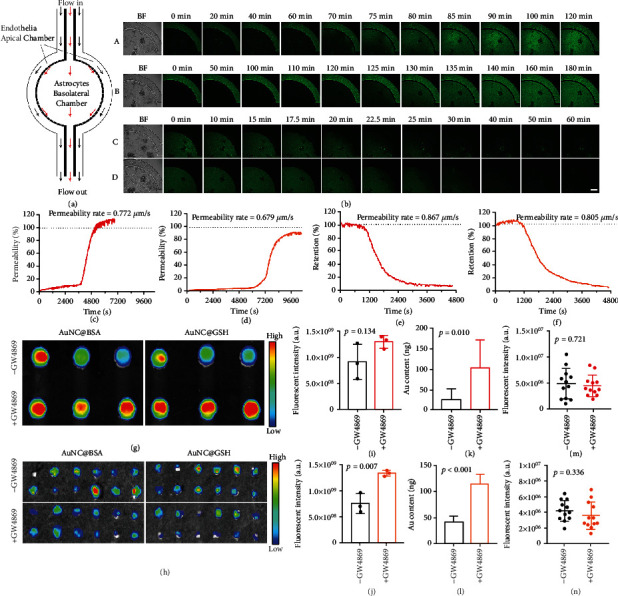
(a) The structure of chip used for the construction of the dynamic BBB model. The brain vascular endothelia grew to interior cover in apical chamber with permanent flow to simulate brain blood vessel, and astrocytes grew in the basolateral chamber with conditional flow (commonly flowed off when experimented) to support the dynamic BBB model, bar represents 200 *μ*m. (b) The real-time fluorescent imaging captured the permeability of AuNCs in the direction of blood to brain (i: AuNC@BSA, ii: AuNC@GSH) or brain to blood (iii: AuNC@BSA, iv: AuNC@GSH), the white bar represents 200 *μ*m, BF = bright field, green fluorescence tracks the FITC on the AuNCs. (c-f) The permeability to time graphs were obtained from a-d, respectively. The permeability rates revealed the efficacy in linear phase. Fluorescent imaging of the WT and GW6849-treated mice brain (g) and CLNs (h), the scale bars range from 2.0 × 10^7^ to 1.2 × 10^8^, and 4.0 × 10^6^ to 1.0 × 10^7^, respectively. The semi-quantification of fluorescence in brain (i, j) and CLNs (m, n), and remaining Au quantified by ICP-MS (k, l) revealed the elimination of AuNCs and glymphatic drainage. The unit for fluorescent imaging is (p/sec/cm^2^/sr)/(*μ*W/cm^2^).

In comparison to the static BBB model created in a transwell chamber, the dynamic BBB model on the chip was more accurate in simulating the permeability of DDS. The endothelium was under a flow in the apical chambers of the chip, and the shear stress would induce the critical tight junction of endothelial layers [58]. The basolateral chamber with astrocytes was commonly static to simulate brain tissue (Figure 6(a)). The results of real-time fluorescence imaging are shown in Figure 6(b). Both AuNC@BSA and AuNC@GSH critically permeated from the apical to basolateral chambers, and their related permeability to time graphs clearly showed a time-dependent manner (Figure 6(c), 6(d)). The AuNCs did not show obvious permeability early on until the first point of inflection (70 min for AuNC@BSA and 120 min for AuNC@GSH), and the endothelium gradually internalized the AuNCs before the permeability was evident (more visible in the videos), indicating that it took time for the passage across the BBB to complete intracellular procedures. Typically, the maximum fluorescence intensity of AuNC@BSA in the basolateral chamber was even stronger than the apical chamber, confirming that it was active transport that carried AuNCs across the BBB model. Importantly, the fluorescence change in the apical chamber was difficult to observe in the direction from the basolateral to apical chambers due to stimulation of the bloodstream by continuous flow. The fluorescence in the basolateral chamber dramatically vanished, demonstrating the permeability of AuNCs. The graphs revealed a similar permeability manner between AuNC@BSA and AuNC@GSH (Figure 6(e), 6(f)), with the AuNCs achieving a point of inflection (20 min) earlier than those in the opposite direction. Except for the point of inflection, all four assays showed a linear phase in permeability, indicating efficient trans-BBB passage. The permeability rates for assays a to d were 0.772, 0.679, 0.867, and 0.805 *μ*m/s, respectively.

The in vivo elimination of AuNCs through BBB was verified using GW6849, an exosomes secretion inhibitor. Exosome secretion by brain endothelial cells was inhibited 21.5 ± 6.9% by GW6849 (Figure S11). After pretreatment with GW6849, the fluorescence of AuNCs in mouse brains was significantly stronger than in WT mice (Figure 6(g)), while the fluorescence in CLNs was comparable (Figure 6(h)). Quantification of fluorescence intensity and Au contents in the brain and CLNs (Figure 6(i)-6(n)) revealed that GW6849 inhibited AuNCs elimination, but did not affect glymphatic drainage, indicating that the exosome-based BBB passage was critical for AuNCs elimination. The inhibition of AuNC@BSA and AuNC@GSH was determined to be 6.6 ± 5.9% and 6.3 ± 1.5%, respectively, while the route inhibition rate was 21.5 ± 6.9%, and the actual contribution to AuNC@BSA and AuNC@GSH elimination through the BBB passage was 30.5 ± 27.3% and 29.2 ± 7.1%, respectively (Table S1).

### 2.7. Microglia Collected AuNCs and Promoted their Glymphatic Drainage

**Figure 7:**
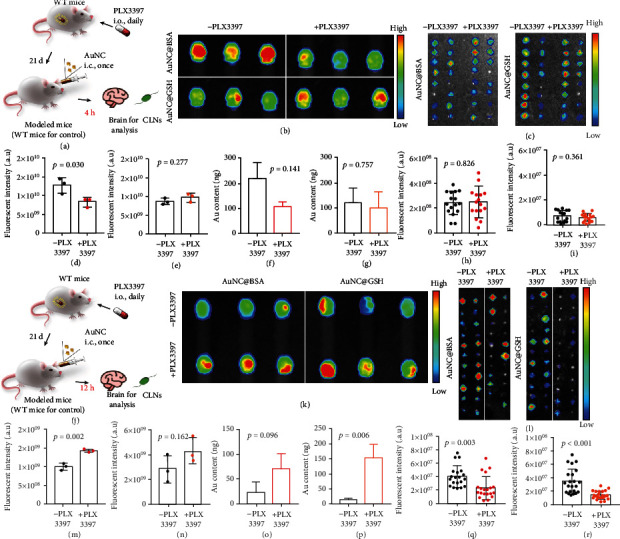
(a) Illustration of the construction of microglia-deficient modeled mice, the mice needed take the PLX3397 daily for a period of 21 days for experiment, and the AuNCs were i.c. injected 4 h (short interval) before the brains and CLNs were collected for analysis. The FLI of the brains (b) and CLNs (c), the color scale of the brain ranges from 1.0 × 10^8^ to 1.0 × 10^9^, and that for the CLNs ranges from 2.0 × 10^7^ to 5.0 × 10^7^. (d-e) The semi-quantification of brain fluorescent intensity. (f-g) The Au contents in the brain measured by the ICP-MS. (h-i) The semi-quantification of the fluorescent intensity in the CLNs. (j) The interval between i.c. AuNCs treatment and samples collection was prolonged to 12 h. The FLI of the brains (k) and CLNs (l), the color scale of the brain received AuNC@BSA ranges from 1.5 × 10^7^ to 1.5 × 10^8^, while 5.0 × 10^7^ to 5.0 × 10^8^ for that of AuNC@GSH. The color scale for the CLNs ranges from 2.0 × 10^7^ to 5.0 × 10^7^. (m-n) The semi-quantification of brain fluorescent intensity. (o-p) The Au contents in the brain measured by the ICP-MS. (q-r) The semi-quantification of the fluorescent intensity in the CLNs. The unit for fluorescent imaging is (p/sec/cm^2^/sr)/(*μ*W/cm^2^).

Since microglia were permanent cells in CNS, we hypothesized that there must be downstream routes that handled the phagocytosed AuNCs in microglia for further elimination. The WT mice were compared to mice treated with pexidartinib (PLX3397), a colony-stimulating factor 1 receptor (CSF-1R) inhibitor, which inhibited the maturation of mouse microglia (Figure 7(a)) [59, 60]. However, 4 h post i.c. AuNCs treatment, there was essentially no difference between the modeled and WT mice that received AuNC@GSH. In contrast, there was a stronger fluorescence intensity in the brains of WT mice receiving AuNC@BSA (Figure 7(b)), which was unexpected. Furthermore, there was no difference between the CLNs obtained from WT and modeled mice (Figure 7(c)). The statistical data of fluorescence intensity (Figure 7(d), 7(e)) and Au contents (Figure 7(f), 7(g)) verified the outcomes, as did the fluorescence intensity of CLNs (Figure 7(h), 7(i)). Confocal fluorescence imaging revealed a promising finding: abundant AuNCs were captured by microglia (Figure S12), and the panoramic scan (Figure S13) showed that the microglia were in an activated state, which reduced its neurites and became spherical [61], with plenty of AuNCs in the cytoplasm. On the other hand, microglia in the modeled mouse brains remained in a resting state, stretching their neurites to monitor the surrounding environment, and lacked phagocytosis capabilities. Therefore, the phagocytosis of microglia may have retained the AuNCs in the brain during the first 4 h post i.c. administration. In contrast, the microglia-deficient condition reduced the retained AuNCs, resulting in the apparent phenomenon that was observed in this study.

The interval between AuNCs treatment and brain collection was prolonged to 12 h (Figure 7(j)), which allowed microglia to traffic the contained AuNCs. Pretreatment with PLX3397 resulted in both AuNCs displaying significantly stronger signals in the brain (Figure 7(k)). Furthermore, the fluorescence of corresponding CLNs differed between modeled and WT mice (Figure 7(l)). The statistical data of fluorescence intensity (Figure 7(m), 7(n)) and Au contents (Figure 7(o), 7(p)) verified the disparities. The fluorescence intensity in CLNs was significantly reduced after pretreatment with PLX3397 (Figure 7(q), 7(r)). A panoramic scan confirmed PLX3397 caused microglia deficiency, and the AuNCs signal was consistent with the data at the organ level (Figure S14). Importantly, microglia in WT mouse brains returned from an activated state to a resting state, indicating that the microglia had completed their contribution. These findings validated that microglia plays a key role in the elimination of AuNCs in the CNS through a time-consuming and glymphatic system-associated pathway.

### 2.8. Microglia Facilitated the AuNCs Leaving the Brain across the BBB

**Figure 8:**
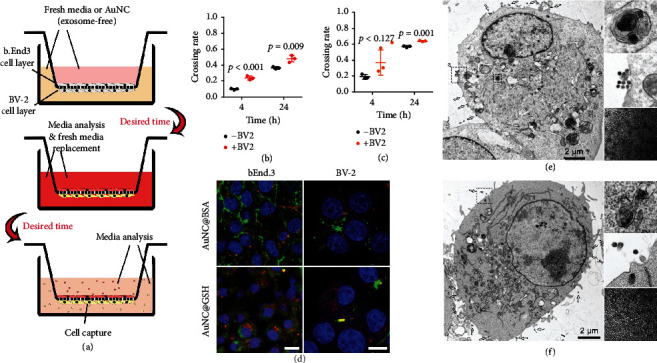
(a) Illustration of the construction of the transwell model to evaluate the potential contribution by the microglia. The AuNCs were always incubated with the cells on the BV-2 cells side, while the post-passage AuNCs were detected on the opposite. The single bEnd.3 cells layer in transwell model were set as control. The permeability of the AuNC@BSA (b) and AuNC@GSH (c) under the condition of with or without BV-2 cells, post 4 or 24 h of incubation. (d) The confocal images of the bEnd.3 and BV-2 cells after incubation with CFSE-labelled AuNCs, the nuclei were showed in blue, red indicated the AuNCs that were just phagocytosed, and the green signal demonstrated the AuNCs that located close to the cytoplasm membrane for subsequent exocytosis, bars represent 5 *μ*m. The TEM captures showed BV-2 cells with sufficient exosomes for the trafficking of AuNC@BSA (e) and AuNC@GSH (f). The arrows indicate the numerous exosomes secreted by the cells. The amplified pictures showed the multivesicular bodies inside the cells, secreted exosomes and the cargo in the exosomes, respectively.

Aside from the glymphatic system, the BBB passage appeared to be the other way that was the downstream pathway of microglia, because microglia can make contact with the BBB [62]. The microglia-involved transwell model (bEnd.3/BV-2, Figure 8(a)) was used to incubate AuNCs in the microglia chamber. When microglia were present, the permeability of AuNCs was significantly enhanced (Figure 8(b), 8(c)), irrespective of whether it was 4 or 24 h. Furthermore, after labeling the AuNCs with carboxyfluorescein succinimidyl amino ester (CFSE), which does not exhibit strong fluorescence until the ester was hydrolyzed in live cells, the freshly phagocytosed AuNCs displayed an obvious red signal in the cytoplasm. However, the AuNCs proceeding for secretion was remarkably green and located near the cytoplasm membrane (Figure 8(d)). Importantly, apart from the AuNCs ready to be secreted, those inside the microglia were pure red, while those inside the endothelium were in orange, suggesting a certain part of the AuNCs had activated green fluorescence before endocytosis by the endothelial cells, indicating intercellular transport of AuNCs. According to a TEM image of microglia, multivesicular bodies containing AuNCs were observed in the cytoplasm. In addition, abundant secretory exosomes were encapsulating 2 nm AuNCs crystals around the cytoplasm membrane were observed (Figure 8(e), 8(f)). However, it should be noted that both AuNCs had an interplanar spacing of 3.08 Å, which corresponded to Au (111) in monoclinic crystals [44, 45]. These results validated that: 1) microglia assisted AuNCs in crossing the BBB for elimination from the CNS, and 2) the facilitation was based on exosome-involved intercellular transport.

## 3. Discussion

Both AuNCs were easy to synthesize using a one-step reduction method. As confirmed by DLS and HRTEM, the synthesized products were uniform, well dispersed, and negatively charged for further use, which was consistent with previous reports [33, 34, 38, 40]. Furthermore, their inner fluorescence and abundant functionalization sites made it easy for them to carry therapeutic agents or dyes, including the FITC that was used in this study. A series of dialysis confirmed that the excess free FITC was completely removed. The labeled AuNCs were systemically distributed in WT mice and were rapidly excreted via the kidney and liver, which was consistent with a previous finding [40]. Meanwhile, the systemic distribution also demonstrated that both AuNCs possessed brain delivery potential, as well as the ability to return the periphery, which was essential for the application of DDS.

The vein-surrounding aggregates of AuNCs found in sub-brain images suggested that brain blood vessels potentially contributed to the process of AuNCs in the CNS, and this phenomenon was consistent with the glymphatic drainage reported in recent years [25, 28–30]. A glymphatic system, by which certain endogenous molecules can leave the CNS, is present. These solutes in ISF generally flow along the basement membrane of capillary veins, reaching the basement membrane between the smooth muscles in the tunica media of arteries, where the subsequent IPAD for solute elimination takes place. Eventually, the solutes reach the periphery by passing through CLNs [63, 64]. The glymphatic system plays a major role in the elimination of brain wastes, including A*β* and other antigens generated in the CNS [30, 65]. However, previous evidence validated that the 15 nm gold nanoparticles accumulated beside the veins but could not be eliminated via the glymphatic system [25]. Therefore, the fate of AuNCs remained unclear, with current information only showing their vein-surrounding position. Fortunately, the TEM images convinced us that the AuNCs were located in the basement membrane of capillaries, which was the passageway occupied by the glymphatic drainage, suggesting that AuNCs may participate in glymphatic drainage to leave the brain.

**Figure 9:**
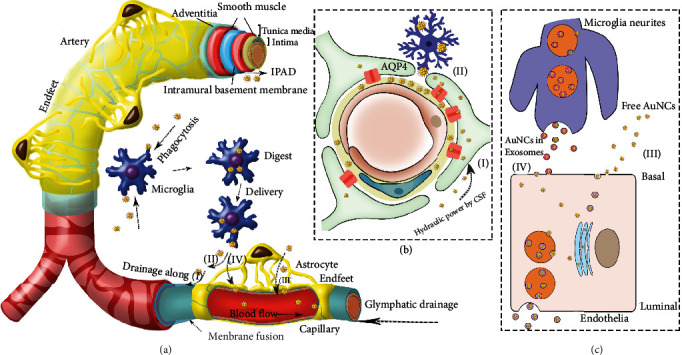
(a) The summary of AuNCs elimination routes from brain is schematically illustrated. The detail procedures of (I) to (IV) are shown in planar graphs and their captions. (b) The glymphatic system stimulated by CSF and AQP4 water channel continuously drained free AuNCs in parenchyma, drove them from capillary basement membrane to arterial basement membrane in tunica media for IPAD (I). The microglia could phagocytose and delivery AuNCs to the glymphatic system for promotion (II). (c) Free AuNCs in parenchyma was accessible to BBB and actively transported across BBB by endocytosis and exocytosis in exosomes (III). The microglia could secrete AuNCs in exosomes to further fuse with endothelia cells, facilitating the transcellular transport (IV).

In order to eliminate the possibility that AuNCs could gather in the basement membrane when entering the brain, which would fabricate the TEM results, the i.c. AuNCs were tracked to evaluate the elimination mechanism. The 3D reconstruction of z-series scanned fluorescence images demonstrated that the AuNCs did travel along the basement membrane in the capillary and artery. Importantly, the occupied arterial basement membrane was located between the smooth muscles in the tunica media, which was convincing evidence for glymphatic drainage of AuNCs (Figure 9(a)). According to previous findings, the ladder-like AuNCs signal in the basement membrane was a typical indication of glymphatic drainage [28, 30]. The TEM images of brains that had received i.c. AuNCs further confirmed the glymphatic drainage of AuNCs and suggested that the AQP4 water channel on the astrocytic end-feet may have allowed AuNCs to enter the glymphatic system.

The dominant glymphatic drainage of AuNCs was considered the major elimination pathway after evaluation of its contribution. The reduction in CSF significantly lowered the drainage rate, demonstrating that glymphatic drainage of AuNCs depended on the hydraulic force of CSF as the motive power. A similar situation occurred when the AQP4 water channel was inhibited. Based on the TEM observation of AuNCs co-localized with astrocytic end-feet, the results synergistically validated that AuNCs accessed the glymphatic drainage through the AQP4 water channel (Figure 9(b)-9(i)). Furthermore, it was worth noting that AuNCs colocalized well with microglia after systemic distribution. Since microglia were permanent cells in the CNS, they must have the ability to deliver the phagocytosed AuNCs to a downstream pathway, which we assumed included the glymphatic system. In order to verify the hypothesis, we inhibited the proliferation and maturation of microglia in the mouse brain and discovered that the long-term elimination of AuNCs was significantly reduced. This reduction was associated with the AuNCs passing through CLNs, revealing the drainage rate of glymphatic system. This finding provided strong evidence that microglia could deliver phagocytosed AuNCs to the glymphatic system for elimination (Figure 9(b)-II). However, when there was limited time, microglia hardly completed gathering, processing, and delivery to the glymphatic system, giving the impression that the microglia did not contribute.

In conclusion, AuNCs could efficiently be eliminated from the brain via the glymphatic system, which was dominant after AuNCs accumulated in the brain, while motive power provided by CSF, AQP4 entrance, and microglia could facilitate the progress of AuNCs glymphatic drainage. Nevertheless, considering the narrow passageway that AQP4 maintained, the thin basement membrane that was occupied by the glymphatic system, as well as a previous report that 15 nm gold nanoparticles could not take advantage of the glymphatic system, there appeared to be a size limit [35] for acceptance by glymphatic drainage, while surficial ligands, charges, and other properties may also limit the progress. Although AuNCs were sufficiently eliminated via the glymphatic system, the universality of the glymphatic system when encountering nanomaterials requires continuous evidence.

Another potential way for AuNCs to leave the brain was by crossing the BBB. However, to our knowledge, there have been no reports of exogenous DDS in nanometers leaving the brain through the BBB, which has been verified to eliminate endogenous wastes, such as A*β* [32]. Furthermore, the passage across the BBB was difficult to capture in vivo due to the confusion caused by the undergoing glymphatic drainage. In addition, determining the precise mechanism proved to be difficult with the numerous disturbances. Therefore, various in vitro models for accurate BBB simulation were helpful to overcome these issues [66–68]. Both static and dynamic BBB models verified that the AuNCs traveled across the BBB structures into the tissue chamber, and their behaviors were consistent with the distribution measured in vivo, demonstrating the practicability of simulation using in vitro BBB models. Meanwhile, critical permeability from the tissue chamber to the vascular chamber demonstrated the potential elimination of AuNCs through the BBB, composing the other clearance mechanism of AuNCs in the CNS. In order to identify the mechanism of BBB passage-involved elimination, the uptake inhibition assays and exosome analysis with TEM capture validated that there were two parts to the processes: 1) AuNCs bound to the receptor or transporter to trigger endocytosis, which further required caveolin and clathrin for motivation, and 2) the intracellular multivesicular bodies trapped AuNCs, further fusing with the cytoplasm membrane to release them in extracellular exosomes for the completion of exocytosis (Figure 9(c)-III).

Apart from the glymphatic system, AuNCs were also eliminated through the BBB, which was a potential downstream pathway through which microglia delivered AuNCs. By using in vitro BBB models, we discovered that microglia promoted the trans-BBB transport of AuNCs. Furthermore, microglia showed stronger endocytosis and subsequent exocytosis of AuNCs involving secretory exosomes, which could fuse with endothelial cells to accelerate transport across the BBB (Figure 9(c)-IV). The series of intercellular transport is potentially the reason why microglia could enhance the AuNCs permeability across BBB.

By taking into account all of the obtained information, the in vivo situation could be figured out: resting microglia stretched their neurites to monitor AuNCs that needed clearance, and once detected, they were activated and performed phagocytosis. Following that, the microglia were processed and, most importantly, cooperated with the BBB to achieve rapid AuNCs elimination, whereas BBB alone undertook a slower elimination of AuNCs. Both types of trans-BBB passages depended on exosome transport, which provided the first glimpse and was essential for DDS delivery. However, since exosomes were extracellular vesicles of around only 100 nm in size, we assumed that there were limitations, such as size, for nanomaterials to be used in this way. Furthermore, surficial modifiers strongly influenced transcellular and intercellular transport. Therefore, more investigations and evidence are required to determine the universality of DDS elimination across the BBB.

## 4. Conclusion

In order to identify the potential elimination routes of DDS post-brain delivery, we investigated two AuNCs to determine the detailed mechanisms. Based on a series of systemic and conditioned distribution analyses, the path through the so-called glymphatic system was the most feasible way for DDS to leave the brain, although exosome transport across the BBB also contributed to DDS elimination. Furthermore, microglia played a key role in collecting the diffused DDS and delivering them for glymphatic drainage or to the BBB passage, significantly facilitating the process. Thus far, the synergistic functions of the glymphatic system, BBB, and microglia were clarified, which not only confirmed the accessibility of DDS leaving the brain but also provided detailed routes and mechanisms. This study provided the first evidence of brain elimination of nano-sized DDS, but more information is required to validate the universality of the theories. In any case, a broader perspective should serve as a critical new guideline for the future development of brain delivery DDS.

## 5. Methods

### 5.1. Gold Nanocluster Synthesis

Both AuNC@BSA and AuNC@GSH were synthesized according to previous reports. All the glassware used for AuNCs synthesis was cleaned using aqua regia, detergent, then deionized water. Firstly, 5 mL of 50 mg/mL BSA (Biofroxx, EZ5679A168) or 15 mM GSH (Meilunbio, MB3281) aqueous solution was added into a round-bottom flask, which was stirred and heated to 37°C. Following that, an equivalent volume of 10 mM chloroauric acid (HAuCl_4_, Sinopharm, 10010711) was added. The mixture was adjusted to pH 12 with 10 M sodium hydroxide (NaOH) 2 min after incubation at 37°C. After a 12-h reaction, the product was obtained after dialysis with deionized water to neutral pH and subsequent filtration with a 0.22 *μ*m membrane (Jinteng, TJMF50). Generally, the AuNC@GSH reaction required around 24 h to yield a dark yellow product. The purified AuNCs were stored at 4°C for further use. Typically for FITC-labelled AuNCs, 0.5 mg of FITC (Aladdin, F106837) was first dissolved in 1 mL of phosphate buffer saline (PBS) solution at pH 9.8. Next, 15 *μ*mol AuNCs (Au content) were mixed with the FITC solution and left to react overnight at room temperature. The excess FITC was completely removed by dialysis until no FITC signal could be detected in the dialysate. Both FITC labeled AuNCs were freshly synthesized before use.

### 5.2. Systemic Distribution

The WT BALC/c mice were i.v. administrated with the two FITC-labelled AuNCs through the tail vein at a dose of 10 mg Au/kg (n =3). The mice were sacrificed at 2, 8, 24 h, and 7 days post-administration, then received successive perfusion of PBS and paraformaldehyde (PFA). Their organs were collected, washed in PBS, and fixed with 4% PFA for at least 24 h. The fluorescence intensity in all ex vivo organs was detected using an in vivo fluorescence imaging system (PerkinElmer, IVIS Lumina III, parameters: EX =480 nm, EM =520 nm, sight of view =12.5 cm, object height =1.5 cm). The region of interest (ROI) was counted using the accompanying software (Living image 4.3). Furthermore, blood samples were obtained at 0.25, 0.5, 3, 6, 12, 24, 36, 48, 60, and 72 h post-injection (n = 5, 50 *μ*L each time). The organs and blood samples were digested with a mixture of 1 mL concentrated nitric acid (HNO_3_) and 0.2 mL hydrogen peroxide (H_2_O_2_) at 110°C overnight for absolute quantification of the Au contents. The apparent light-yellow solution was filtered through a 0.22 *μ*m membrane and diluted in 2% HNO_3_ to a suitable Au concentration (0-20 ppb). The aqueous samples were detected using ICP-MS (PerkinElmer, NexIon 350x) and the Au contents in the organs or blood were recalculated.

For tissue slices, the fixed organs were dewatered by successively soaking them in 15% and 30% sucrose solution for 24 h, then a 3 mm flake of each organ was embedded in the tissue freezing medium (Sakura, tissue-tek 4583). A freezing microtome (Leica, CM1950) was used to obtain 10 *μ*m thin tissue slices, which were washed with PBS three times, stained with DAPI (5 *μ*g/mL) for 5 min to track the nuclei, then mounted on cover glasses. In order to label the blood vessels, the tissue slices were washed with PBS, blocked with goat serum for 1 h, and incubated with rabbit anti-CD31 antibody (Abcam, ab324774, 1 : 200) at 4°C overnight. Following that, the tissue slices were incubated with Alexa Fluor 647 conjugated goat-anti-rabbit IgG (Invitrogen, 2686730, 1 : 500) for 1 h at room temperature. Immunofluorescence labeling of microglia or astrocytes was carried out as previously described using rabbit anti-TMEM-119 antibody (Abcam, ab209064, 1 : 200) or rabbit anti-GFAP antibody (Abcam, ab207165, 1 : 500). All fluorescence slices were imaged using a confocal fluorescence microscope (Nikon, NIS-Elements A1). For TEM capture, around 3 mm^3^ fresh brain pieces were obtained and soaked in 2.5% glutaraldehyde for at least 24 h. The fixed brain pieces were then stained with lead citrate and sectioned into ultrathin slices for TEM imaging.

### 5.3. Distribution of AuNCs in the Brain Post I.c. Injection

The FITC-labeled AuNCs were neutralized, isosmotically adjusted, and diluted to 3 mM. The WT BALB/c mice were fastened by a stereotaxic apparatus, and their scalp was cut open to allow the erosion of the tissue covering the skull. The microsyringes were then moved to a hippocampal site (1.8 mm right and 2.3 mm backward from the bregma). A volume of 2 *μ*L of the FITC-labelled AuNCs was injected into the site 2 mm below the skull at a rate of 0.4 *μ*L/min. Time was recorded from when the injection was completed, and the microsyringes were retained for 5 min to avoid overflow. The mice were sacrificed at 5- or 30-min post-injection. Their brains were collected fresh for fixation, then sectioned into ultrathin or immunofluorescence slices as described above. The embedded dewatered brains were sectioned into 100 *μ*m slices, which were then co-labeled with mouse eFluor 660-conjugated anti-*α*-SMA antibody (eBioscience, 50-9760-82, 1 : 500) and rabbit anti-collagen IV antibody (Abcam, ab236640, 1 : 300), with the latter being marked by Alexa Fluor 405-conjugated donkey-anti-rabbit IgG (Abcam, ab175651, 1 : 500). The primary antibody incubation period was prolonged to 24 h, while the secondary antibody was allowed to incubate for 4 h. The 100 *μ*m thick slices were scanned in z-series (2.5 *μ*m per step for about 50 *μ*m) for confocal imaging, then the Z-series images were reconstructed into a 3D image using the NIS-Elements Analysis software. Videos that displayed the 3D structure were created using artificial rotation and recording (20 s for a 360° rotation).

### 5.4. Inhibition of AuNCs Elimination In Vivo

In order to obtain CSF-reduced mice, ACZ (Aladdin, A194116) was intraperitoneally administered at a dose of 20 mg/kg/time four times with a 6 h interval. The modeled mice were subjected to the experiment 2 h after the last injection. The AQP4-inhibited mice were created by intraperitoneal injection of 100 mg/kg TGN-020 (InvivoChem, V4001) 15 min before the experiment. GW6849 (AdooQ, A11974) was used to inhibit in vivo exosome secretion at a dose of 2.5 mg/kg (i.p.), and the modeled mice were subjected to the experiment 1 h later. The microglia-deficient mice were obtained by daily intraoral administration of PLX3397 (AdooQ, A15520) at 40 mg/kg/time for 21 days, with the experiment carried out 4 h after the last injection. All modeled or WT mice were injected with 2 *μ*L of the FITC-labelled AuNCs as described above. The brains and CLNs in the CSF and AQP4 inhibition experiments were collected 2 h post the injection of the AuNCs, while the intervals for the two microglia-inhibition experiments were 4 and 12 h, respectively. As with the methods described above, FLI and ICP-MS were used to analyze the contents of AuNCs in brains and CLNs. In order to label the peri-artery macrophages and microglia in the brains, the rabbit anti-CD163 antibody (Abcam, ab182422, 1 : 200) and rabbit anti-Iba-1 antibody (Abcam, 178846,1 : 500) were used, with the Alexa Fluor 647 conjugated goat-anti-rabbit IgG (Invitrogen, 2686730, 1 : 500) used as the secondary antibody. In addition to confocal imaging, the microglia-labeled slices were also subjected to panoramic scanning.

### 5.5. Permeability in Transwell BBB Models

For co-incubated BBB models, the insert chambers of the transwell plate (NEST, TC724001, and TC725001) were initially placed upside down in a dish with sufficient PBS to seal the edge. Sterile silicone tubes in suitable sizes were then used to circle grasp the bottom. Subsequently, U251 or BV-2 cells in media were added to the silicon tube to cover the membrane, and incubated for 12 h to allow the cells to attach. The bEnd.3 cells were seeded on the other side of the membrane after the insert chambers were reset in the plate. The two types of cells were allowed 48 h to co-attach and communicate. The densities of the bEnd.3, U251, and BV-2 cells used for the BBB models were 3 × 10^5^, 1 × 10^5^, and 1 × 10^5^ cells per well (for 12-well plates, one-third for 24-well plates), respectively. The BBB model, with a single layer of bEnd.3 cells, was created using the same method without pre-seeding the U251 or BV-2 cells. The transepithelial electrical resistance (TEER) was measured daily and was required to be larger than 180*Ω*·cm^2^ before it could be used. The AuNCs (0.2 mM Au, same as in vitro incubation) were introduced into the insert chamber or bottom chamber of different transwell BBB models, with the opposite chamber full of fresh medium. The medium samples were collected for analysis using a microplate reader (Multiskan MK3, Thermo, USA) at specified times.

### 5.6. The Construction and Permeability Assay of the BBB on a Chip

The detailed procedure of creating a dynamic BBB model was carried out according to the accompanying instructions. In brief, the obtained chips (SynVivo, USA) were filled with human fibronectin (200 *μ*g/mL, Sigma, F0895) in the chambers, and a purge pump with sterile gas was used to remove bubbles in the chambers under 5-7 psi. The bubble-free chips were incubated for 1 h at 37°C. For the cells in the chambers, cell suspensions (5 × 10^6^ cells/mL for endothelial cells and 2 × 10^7^ cells/mL for astrocytes) were injected into relevant chambers using a syringe pump (Longer, LSP10-1B) at a flow rate of 5 *μ*L/min. The media was replenished using a syringe pump at a flow rate of 2 *μ*L/min for 3 min, with the frequency (varying from every 12 h to every 3 h) dependent on cell density. When the endothelial cells were confluent, a gradually increasing flow was used to induce a tight junction to the dynamic BBB model before the experiments were carried out according to the manufacturer's instructions. For the permeability analysis, the FITC-labeled AuNCs were introduced separately into the relevant chambers, and fluorescence imaging was performed every 30 s until sufficient permeability was achieved. Semi-quantification was carried out using the Image J software according to the manufacturer's instructions, and the permeability rate was calculated from the following equation:

(1) $$\begin{array}{c} \text{P}=\left(1-{\text{H}}_{\text{ct}}\right)\left({\text{I}}_{\text{t}}/{\text{d}}_{\text{t}}\right)\left(\text{V}/\text{S}\right)/{\text{I}}_{0}, \end{array}$$

where H_ct_ represented the hematocrit count (which was 0 in vitro), V/S represented the volume to surface ratio of the apical chamber and was specified as 0.1 cm in the instruction. I_0_ represented the initial fluorescence intensity of the apical or basolateral chambers, and the I_t_/d_t_ represented the slope of fluorescence intensity change in the permeable chamber.

### 5.7. Exocytosis by Endothelial Cells and Promotion by Microglia

The bEnd.3 and bEnd.3/BV-2 models were incubated with AuNCs at the basal side of bEnd.3 for 1 h, after which the entire medium was replenished for the next 0.5 h incubation. The cells were collected for TEM imaging, and the medium was also collected for exosome analysis. The cells in suspension were centrifuged at 2000 g for 3 min in a 1.5 mL tube, then the supernatant was completely removed and the cell pellet was saturated in 2.5% glutaraldehyde for sufficient fixation. The fixed cell pellet was stained and sectioned into ultrathin slices, which were required for the TEM imaging. Exosomes in the medium were extracted and purified through a general procedure. The cell medium was centrifuged at 3000 g for 15 min, then at 4000 g for 30 min to completely remove potential cells and fragments. Next, a half volume of exosome extractor (Invitrogen, 4478359) was added to obtain a good mixture, followed by incubation at 4°C overnight and centrifugation at 10000 g for 1 h. Exosomes were obtained by resuspending the invisible pellet at the bottom of the tube. The extraction was repeated for purification. The blank exosomes were incubated with a FITC-labeled rabbit anti-CD9 antibody (BD, 341636) for 10 min at room temperature, then the labeled exosomes were resuspended in 20× volume of PBS and reextracted for purification. The purified exosomes were analyzed using the nanoFCM (NanoFCM, N30E). Purified exosomes containing AuNCs were analyzed using the nanoFCM without staining. For confocal imaging of the potential interaction between BV-2 and bEnd.3 cells, CFSE was used to modify the AuNCs, which were incubated with the BV-2/bEnd.3 model on the BV-2 side. Post 1 h of incubation, both the two cells were collected for slices. The slices were washed with PBS, stained with DAPI (5 *μ*g/mL) for 5 min, and then mounted on cover glasses. Images were acquired with a confocal fluorescence microscope.

## Data Availability

All data supporting the findings in this study are available from the corresponding author (Huile Gao, gaohuile@scu.edu.cn) upon reasonable request.

## Supplementary Materials

Supplementary Materials Supplementary method provide additional methods including materials characterization, cell culture and animal maintain, etc, used in this study. Supplementary table: the contributions of glymphatic drainage and BBB passage to AuNCs elimination. Supplementary figures: Figure S1: Systemic distribution of AuNCs. Figure S2: Semi-quantification of the AuNCs fluorescence intensity in brain. Figure S3: ICP-MS analysis of Au in brain. Figure S4: AuNC@BSA distribution in tissue. Figure S5: AuNC@GSH distribution in tissue. Figure S6: Co-localization of AuNCs and blood vessels. Figure S7: ACZ treatment inhibited inulin elimination from brain. Figure S8&S9: Confocal images of peri-artery macrophages phagocytosing AuNCs after treatment. Figure S10: Nano flow cytometer analysis of the CD9 expression on endothelia exosomes. Figure S11: GW4869 inhibited the exosomes secretion by endothelia cells. Figure S12: Confocal images of labelled microglia phagocytosing AuNCs. Figure S13&S14: Panoramic scan of brain shows the remaining AuNCs and microglia state. Supplementary videos: SI-glymphatic drainage-AuNC@BSA-5 min: 3D view of brain glymphatic system draining AuNC@BSA 5 min post i.c. injection. SI-glymphatic drainage-AuNC@BSA-30 min: 3D view of brain glymphatic system draining AuNC@BSA 30 min post i.c. injection. SI-glymphatic drainage-AuNC@GSH-5 min: 3D view of brain glymphatic system draining AuNC@GSH 5 min post i.c. injection. SI-glymphatic drainage-AuNC@GSH-30 min: 3D view of brain glymphatic system draining AuNC@GSH 30 min post i.c. injection. SI-BBB chips-AuNC@BSA-in: Fluorescence imaging of AuNC@BSA transport from blood vessels to brain parenchyma on BBB chip. SI-BBB chips-AuNC@BSA-out: Fluorescence imaging of AuNC@BSA transport from brain parenchyma to blood vessels on BBB chip. SI-BBB chips-AuNC@GSH-in: Fluorescence imaging of AuNC@GSH transport from blood vessels to brain parenchyma on BBB chip. SI-BBB chips-AuNC@GSH-out: Fluorescence imaging of AuNC@GSH transport from brain parenchyma to blood vessels on BBB chip.
