# Supplementary material for: Glymphatic System and Subsidiary Pathways Drive Nanoparticles Away from the Brain
Source: Research (Wash D C). 2022 Mar 15;2022:9847612. doi: 10.34133/2022/9847612 (PMC8943630; doi:10.34133/2022/9847612)
Supplement: Supplementary Materials — Supplementary method provide additional methods including materials characterization, cell culture and animal maintain, etc, used in this study. Supplementary table: the contributions of glymphatic drainage and BBB passage to AuNCs elimination. Supplementary figures: Figure S1: Systemic distribution of AuNCs. Figure S2: Semi-quantification of the AuNCs fluorescence intensity in brain. Figure S3: ICP-MS analysis of Au in brain. Figure S4: AuNC@BSA distribution in tissue. Figure S5: AuNC@GSH distribution in tissue. Figure S6: Co-localization of AuNCs and blood vessels. Figure S7: ACZ treatment inhibited inulin elimination from brain. Figure S8&S9: Confocal images of peri-artery macrophages phagocytosing AuNCs after treatment. Figure S10: Nano flow cytometer analysis of the CD9 expression on endothelia exosomes. Figure S11: GW4869 inhibited the exosomes secretion by endothelia cells. Figure S12: Confocal images of labelled microglia phagocytosing AuNCs. Figure S13&S14: Panoramic scan of brain shows the remaining AuNCs and microglia state. Supplementary videos: SI-glymphatic drainage-AuNC@BSA-5 min: 3D view of brain glymphatic system draining AuNC@BSA 5 min post i.c. injection. SI-glymphatic drainage-AuNC@BSA-30 min: 3D view of brain glymphatic system draining AuNC@BSA 30 min post i.c. injection. SI-glymphatic drainage-AuNC@GSH-5 min: 3D view of brain glymphatic system draining AuNC@GSH 5 min post i.c. injection. SI-glymphatic drainage-AuNC@GSH-30 min: 3D view of brain glymphatic system draining AuNC@GSH 30 min post i.c. injection. SI-BBB chips-AuNC@BSA-in: Fluorescence imaging of AuNC@BSA transport from blood vessels to brain parenchyma on BBB chip. SI-BBB chips-AuNC@BSA-out: Fluorescence imaging of AuNC@BSA transport from brain parenchyma to blood vessels on BBB chip. SI-BBB chips-AuNC@GSH-in: Fluorescence imaging of AuNC@GSH transport from blood vessels to brain parenchyma on BBB chip. SI-BBB chips-AuNC@GSH-out: Fluorescence imaging of AuNC@GSH transport from brain p [file 9847612.f1.zip › brain elimination route-supporting information.docx]

***Supporting information for***

**Glymphatic system and subsidiary pathways drive nanoparticles away from the brain**

*Rui Liu, Yushan Wang, Wenfeng Jia, Chuan Hu, Wenqi Yu, Yuan Huang, Ling Wang and Huile Gao**

Key Laboratory of Drug-Targeting and Drug Delivery System of the Education Ministry and Sichuan Province, Sichuan Engineering Laboratory for Plant-Sourced Drug and Sichuan Research Center for Drug Precision Industrial Technology, West China School of Pharmacy, Sichuan University, Chengdu, 610041, China

*Corresponding author: gaohuile@scu.edu.net

***Supplementary method***

*Particle characterization*

The two AuNCs were freshly made when applied for DLS (Malvern ZS90) and TEM (FEI, Tecnai G2 F20 S-TWIN) measurements. For DLS analysis, the AuNCs were in deionized water at a concentration of 3 mM Au. The TEM and HRTEM images were captured post diffusing the AuNCs on the copper meshes and dryness, without any staining.

*Animal maintain*

BALB/c mice (Byrness Weil biotech Ltd, 18-20 g, 2-4 weeks old) were raised under the guidelines evaluated and approved by the ethics committee of Sichuan University. The animals were housed at the temperature around 22-25°C, and humidity of 50%. The food and water were kept sufficient, and held a day/night cycle of 14/10 hours. For all the animal experiments, each cage maintained 3 mice in one group, avoiding potential disturbance between different groups of mice.

*Cell culture*

The primary bEnd.3 and U251 cells were purchased from Huaer Biotech Co., Ltd (Wuhan), and BV-2 cells were obtained from the Chinese Academy of Sciences Cells Bank (Shanghai). For regular culture, the cells were maintained in an incubator under 37°C and 5.0% CO_2_. High glucose dulbecco’s modified eagle medium (DMEM) with fetal bovine serum (FBS, 10%, Gibco, 10270-106) and penicillin/streptomycin (1%, Solarbio, p1400).

*The uptake mechanism of the endothelia cells*

To investigate the uptake mechanism of bEnd.3 cells, the basal side of bEnd.3 cells in the transwell model was pretreated with fresh medium, 1.3 mg/mL M-beta-CD, 3.5 μg/mL chlorpromazine, 50 μg/mL genistein, 100 μg/mL amiloride and 1 μg/mL colchicine, respectively, for 1 h. Then the AuNCs was also incubated at the basal side, and taking another 1 h for uptake. The cells were collected and repeatedly centrifuged at 2000 g for 3 min and suspended in PBS to purify, and analyzed by a flow cytometer (Agilent, NovoCyte 2060R). The competitive inhibition was carried out similarly with pretreatments of different concentrations of free BSA or GSH.

*Contribution of glymphatic system and BBB passage*

FITC-inulin was used as a standard material to measure the inhibition rate of glymphatic system. Typically, 2 μL of 1 mg/mL FITC-inulin was i.c. injected in ACZ-treated and WT mice, and the organs and CLNs were collected 2 h later. FLI was employed to detect the remaining FITC-inulin. For accurate quantification, the brain samples were homogenized with 9-fold PBS and diluted into 6 mL, microplate reader (Multiskan MK3, Thermo, USA) was used to quantify the remaining FITC-inulin. To measure the inhibition rate of exosomes secretion by brain endothelia cells, 10 μM GW6849 in exosomes-free culture media was used to incubate with b.End3 cells for 4 h, and the media was collected for exosomes extraction. BCA assay kit was employed to quantify the secretory exosomes. For both the two routes, the contribution was calculated as

Contribution = R_Inhibition of AuNCs_ / R_Inhibition of Routes_

*Data analysis and statistics*

Statistics analysis of the data obtained in all the experiments were made by using Graph Pad Prism 7.0. Data involved in the fluorescence imaging were calculated by Living image 4.3. The results of nanoFCM were analyzed by FlowJo 7.6. All the pictures showing the results were optimized and processed by Photoshop CS6. Two-tailed unpaired t-tests was used to compare the means of two dependent groups and find out whether there was a significant difference between them.

***Supplementary table***

Table 1. The contribution of glymphatic system and BBB passage for the elimination of AuNCs.

|  | R_Inhibition of AuNCs_ | R_Inhibition of Routes_ | Contribution |
| --- | --- | --- | --- |
| AuNC@BSA  (glymphatic system) | 19.7±5.9% | 25.4±0.8% | 77.8±23.2% |
| AuNC@GSH  (glymphatic system) | 11.1±5.9% | 25.4±0.8% | 43.7±23.4% |
| AuNC@BSA  (BBB passage) | 6.6±5.9% | 21.5±6.9% | 30.5±27.3% |
| AuNC@GSH  (BBB passage) | 6.3±1.5% | 21.5±6.9% | 29.2±7.1% |


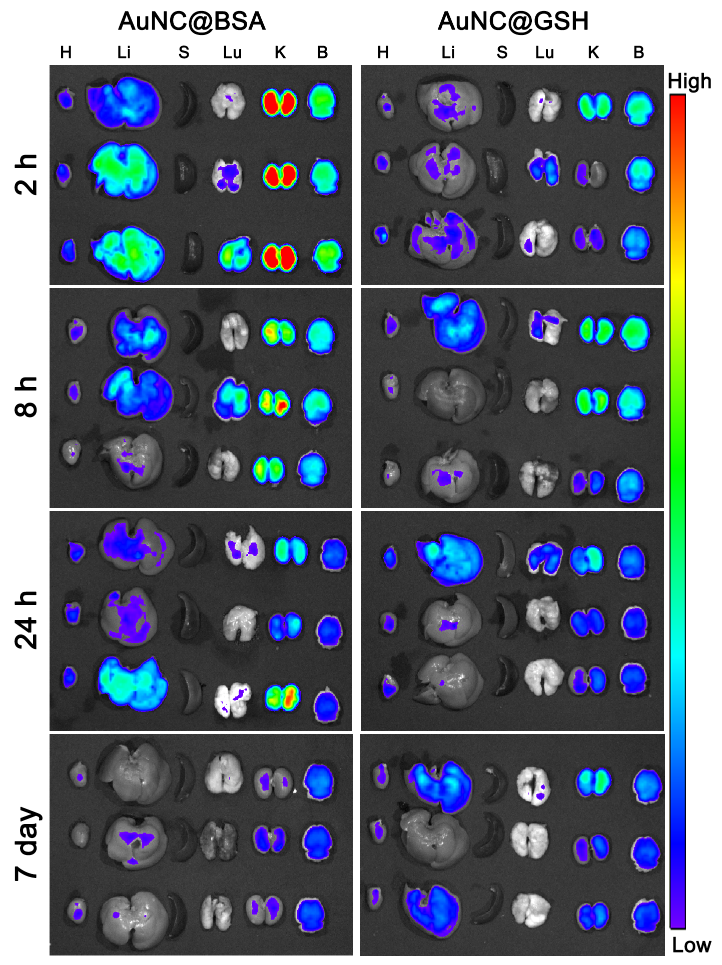


Figure S1. The systemic distribution of AuNCs in the organs of WT mice measured by FLI, The color scale ranges from 1×10^8^ to 6×10^8^, and unit is (p/sec/cm^2^/sr) / (μW/cm^2^), H = heart, Li = liver, S = spleen, Lu = lung, K = kidney, B = brain.


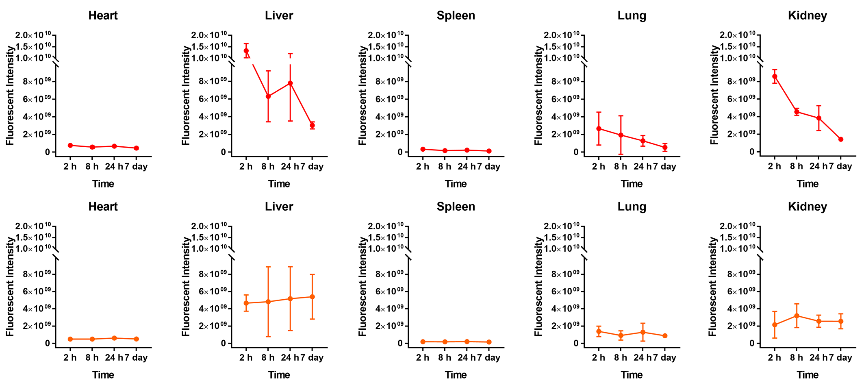
Figure S2. The semi-quantification of fluorescent intensity in organs revealed the distribution of AuNCs in WT mice.


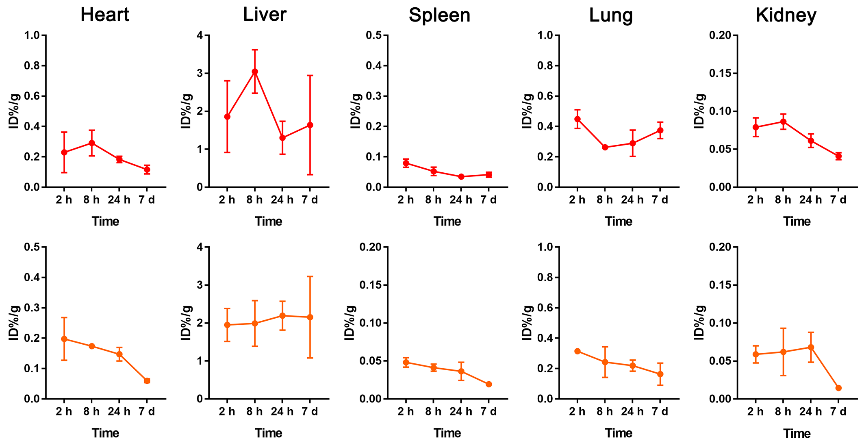
 Figure S3. The ICP-MS measurements of Au accumulation in organs post the systemic distribution of AuNCs.


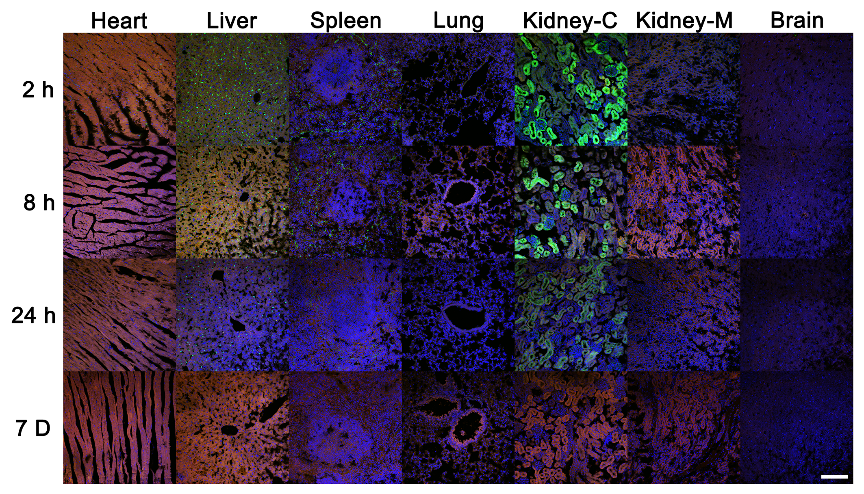
 Figure S4. The confocal fluorescent imaging of AuNC@BSA distribution in sub-organs level, blue = nuclei, green = FITC on AuNC shell, red = AuNC core, kidney-C = the cortex of kidney, kidney-M = the medulla of kidney (the same below), the white bar = 100 μm.


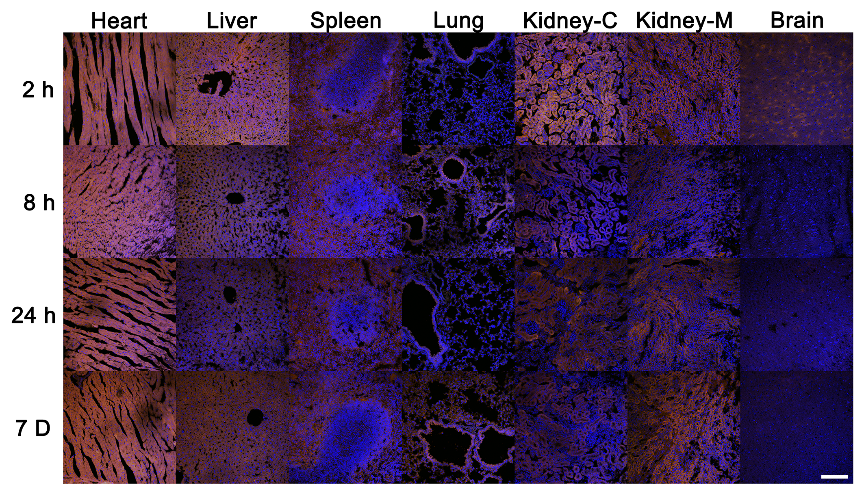
 Figure S5. The confocal fluorescent imaging of AuNC@BSA distribution in sub-organs level, blue = nuclei, green = FITC on AuNC shell, red = AuNC core, the white bar = 100 μm.


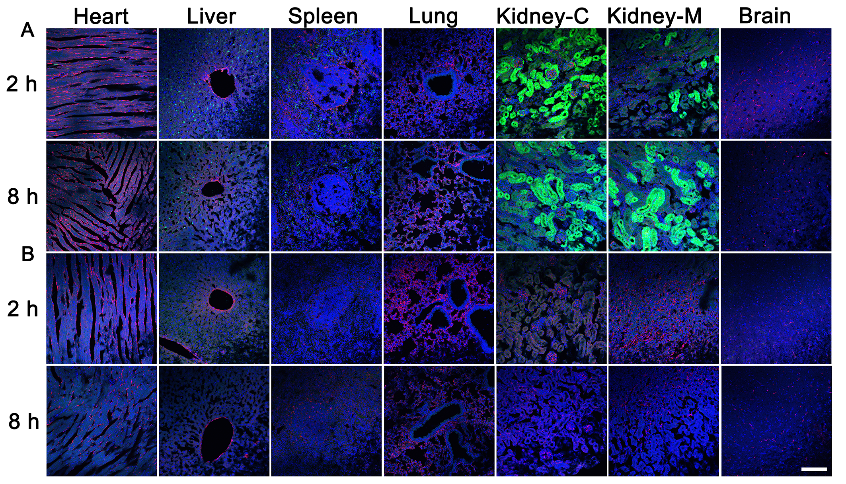


Figure S6. The confocal fluorescent imaging the sub-organs location of AuNC@BSA (A) and AuNC@GSH (B) with blood vessels, blue = nuclei, green = AuNCs, red = blood vessel, bar = 100 μm.


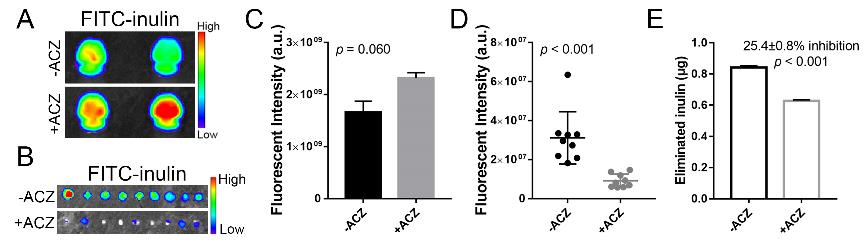


Figure S7. Fluorescent imaging of WT and ACZ-treated mice brain (A) and CLNs (B), the scale bars range from 2.0×10^7^ to 2.0×10^8^, and 1.5×10^7^ to 4.0×10^7^, respectively. The corresponding fluorescent intensity in brain (C) and CLNs (D). (E)The quantification of absolute remaining FITC-inulin in mice brain.


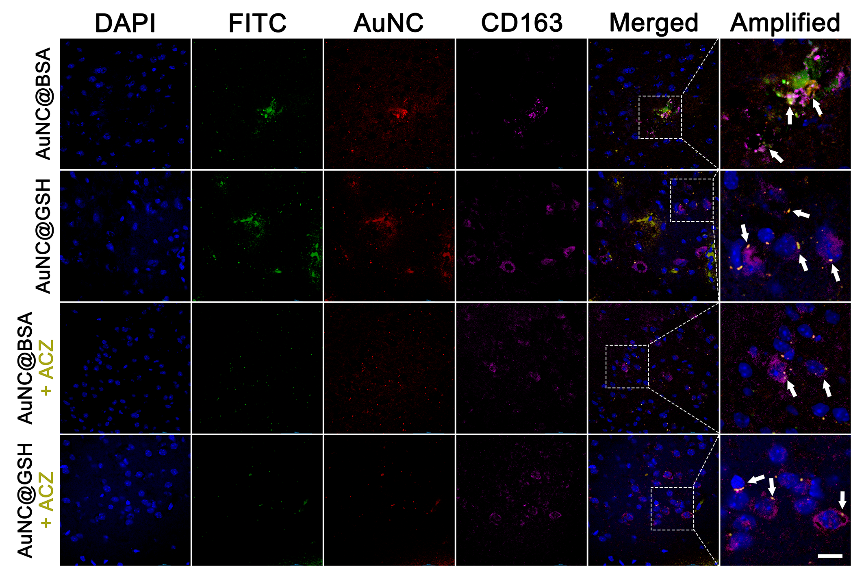
 Figure S8. The confocal fluorescent imaging of brain tissue in WT and ACZ-treated mice, with the collocation of CD163-labelled peri-artery macrophages and phagocytosed AuNCs, bar = 5 μm.


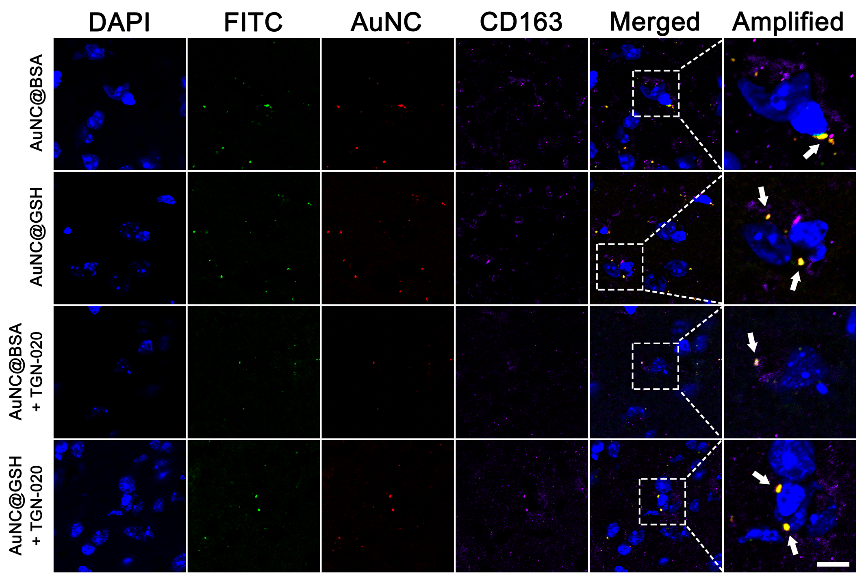
 Figure S9. The confocal fluorescent imaging of brain tissue in WT and TGN-020-treated mice, with the collocation of CD163-labelled peri-artery macrophages and phagocytosed AuNCs, bar = 5 μm.


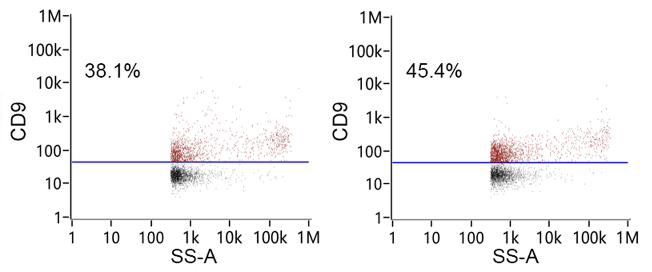


Figure S10. Nano-FCM analyzed the CD9 expression on the exosomes obtained from the luminal (left) and basal side (right) of b.End3 cells.


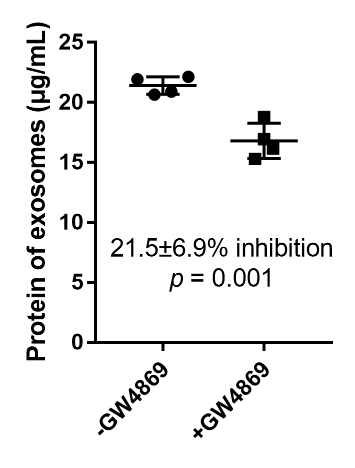


Figure S11. The exosomes secretion of brain endothelia cells was inhibited by GW6849.


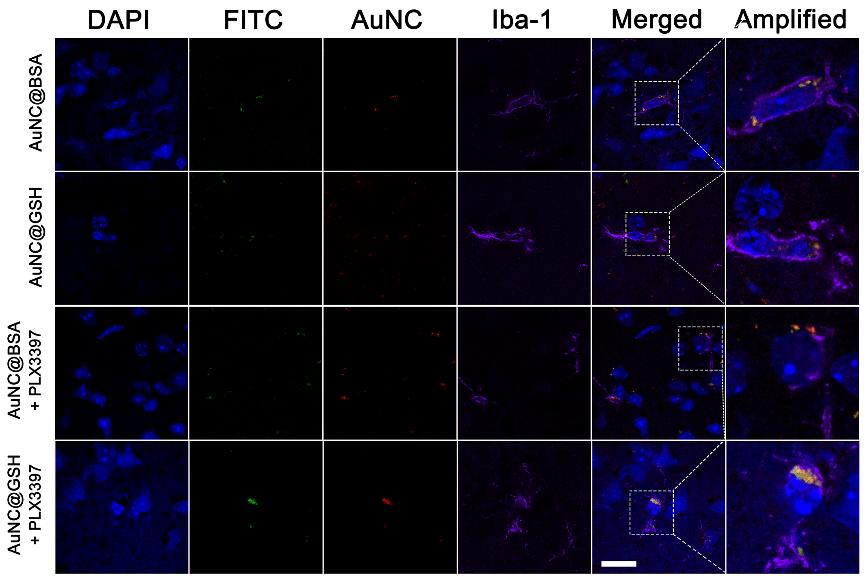
 Figure S12. The confocal fluorescent imaging of brain tissue in WT and PLX3397-treated mice, tracking the Iba-labelled microglia and the retained AuNCs 4 h post i.c. injection, bar = 10 μm.


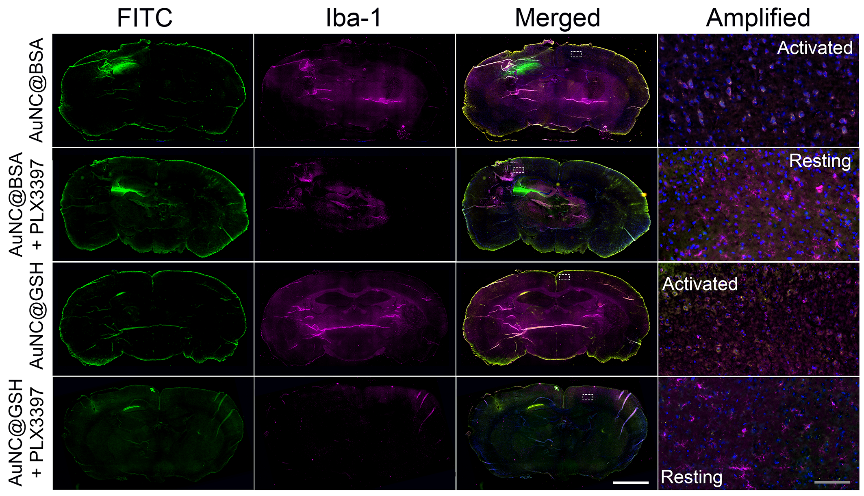


Figure S13. The panoramic scan of the whole brain with AuNCs (green and red) and microglia (pink) labelled (4 h post-injection), nuclei were in blue. The white bar represents 2 mm, and the grey bar represents 100 μm.


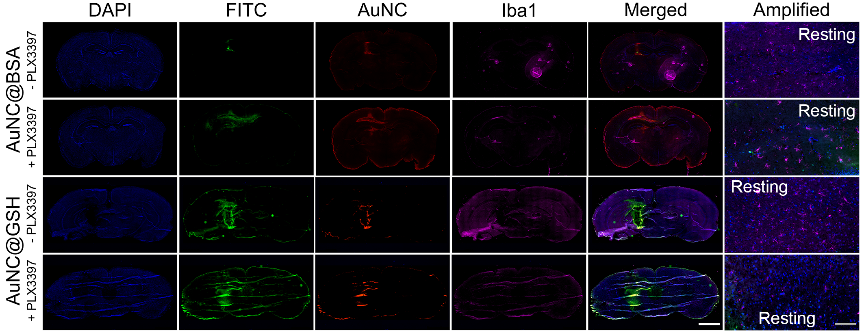


Figure S14. The panoramic scan of the whole brain with AuNCs (green and red) and microglia (pink) labelled (12 h post-injection), nuclei were in blue. The white bar represents 2 mm, and the grey bar represents 100 μm.
